# Supplementary material for: Netcombin: An algorithm for constructing optimal phylogenetic network from rooted triplets
Source: PLoS One. 2020 Sep 18;15(9):e0227842. doi: 10.1371/journal.pone.0227842 (PMC7500971; doi:10.1371/journal.pone.0227842)
Supplement: S4 File — (ZIP) [file pone.0227842.s004.zip › SequenceData-Triplet/sequenceData-10-20-30-0-Triplet/length 300, numseq 40, rate 10^-7/5 seq.pdf]

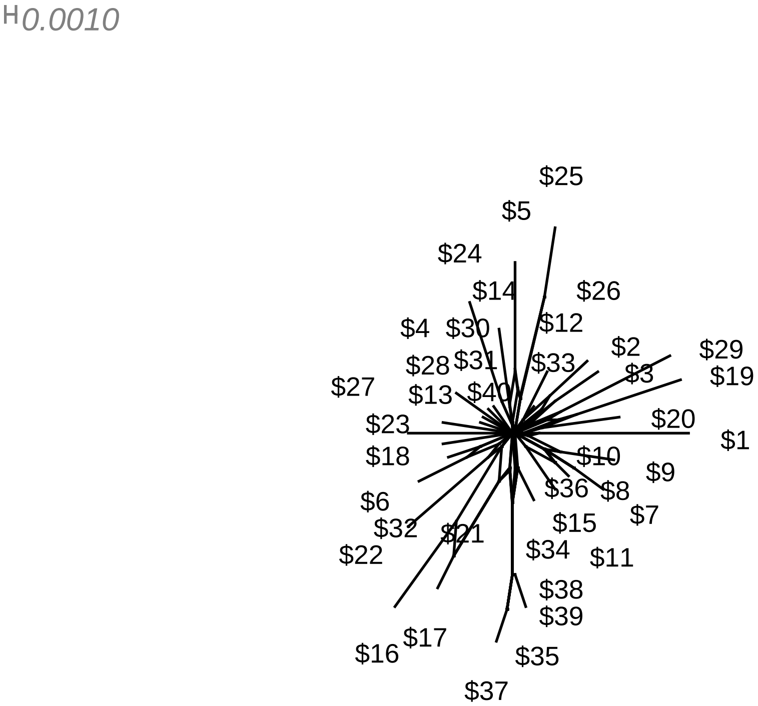
0.0010
$25
$5
$24
$14
$26
$4
$30
$12
$2
$29
$28
$31
$33
$3
$19
$27
$13
$40
$23
$20
$1
$18
$10
$9
$36
$8
$6
$7
$32
$15
$21
$34
$22
$11
$38
$39
$17
$16
$35
$37
